# Association between Previous CPAP and Comorbidities at Diagnosis of Obesity-Hypoventilation Syndrome Associated with Obstructive Sleep Apnea: A Comparative Retrospective Observational Study

**DOI:** 10.3390/jcm12072448

**Published:** 2023-03-23

**Authors:** Moustapha Agossou, Berenice Awanou, Jocelyn Inamo, Marion Dufeal, Jean-Michel Arnal, Moustapha Dramé

**Affiliations:** 1 Department of Respiratory Medicine, University Hospital of Martinique, 97200 Fort-de-France, France; 2 Department of Cardiology, University Hospital of Martinique, 97200 Fort-de-France, France; 3 Service de Réanimation Polyvalente, Hôpital Sainte Musse, 83100 Toulon, France; 4 Department of Clinical Research and Innovation, University Hospital of Martinique, 97200 Fort-de-France, France; 5 EpiCliV Research Unit, Faculty of Medicine, University of the French West Indies, 97261 Fort-de-France, France

**Keywords:** obesity-hypoventilation syndrome, obstructive sleep apnea, CPAP, comorbidities, cardiovascular diseases

## Abstract

Obesity-hypoventilation syndrome (OHS) is associated with many comorbidities. The aim of this study was to evaluate the association between previous continuous positive airway pressure (CPAP) and the prevalence of comorbidities in OHS associated with obstructive sleep apnea (OSA). We performed a retrospective, single-center study at the University Hospital of Martinique, the referral hospital for the island of Martinique. A total of 97 patients with OHS associated with severe OSA on non-invasive ventilation (NIV) were included; 54 patients (56%) had previous treatment of OSA with a positive airway pressure (PAP) device before shifting to NIV (PAP group) and 43 (44%) had no previous treatment of OSA with a PAP device before initiating NIV PAP (no PAP group). Sociodemographic characteristics were similar between groups; there were 40 women (74%) in the PAP group versus 34 (79%) in the no PAP group, mean age at OHS diagnosis was 66 ± 15 versus 67 ± 16 years, respectively, and the mean age at inclusion 72 ± 14 versus 71 ± 15 years, respectively. The average number of comorbidities was 4 ± 1 in the PAP group versus 4 ± 2 in the no PAP group; the mean Charlson index was 5 ± 2 in both groups. The mean BMI was 42 ± 8 kg/m^2^ in both groups. The mean follow-up duration was 5.8 ± 4.4 years in the PAP group versus 4.7 ± 3.5 years in the no PAP group. Chronic heart failure was less common in patients who had a previous PAP 30% versus 53% (*p* = 0.02). It is also noted that these patients were diagnosed less often in the context of acute respiratory failure in patients with previous PAP: 56% versus 93% (*p* < 0.0001). In contrast, asthma patients were more frequent in patients with previous treatment of OSA with a PAP device at the time of OHS diagnosis but not significantly: 37% versus 19% (*p* = 0.07). Early treatment of severe OSA with a PAP device prior to diagnosis of OHS seems to be associated with a reduced prevalence of cardiac diseases, notably chronic heart failure, in patients diagnosed with OHS associated with severe OSA.

## 1. Introduction

Obesity-hypoventilation syndrome (OHS) is a respiratory complication of obesity associated with significant morbidity and mortality, as well as considerable utilization of healthcare resources [1]. Obstructive sleep apnea (OSA) is often associated with OHS. Indeed, OHS is associated with many metabolic and cardiovascular comorbidities. These comorbidities have a major impact on the course of the disease, to the point where it has been proposed to integrate the presence of cardiometabolic comorbidities at the most severe stage of OHS [2]. Management of OHS was recently codified with a recommendation for first-line stable positive airway pressure (PAP) therapy, and a subsequent switch to non invasive ventilation (NIV) in case of PAP failure [3]. Many obese patients with OSA will develop OHS, while patients with OHS on PAP may need a switch to NIV. Several studies have investigated the impact of PAP on comorbidities, but the majority of investigations were performed in patients with OSA [4,5,6].

Regarding the cardiovascular effects, prospective studies have evaluated the effect of continuous positive airway pressure (CPAP) on heart rhythm, arterial hypertension and cardiac biomarkers [5,6]. The metabolic effects investigated include mainly insulin resistance and the effects of CPAP on glucose metabolism, as determined by an oral glucose tolerance test [4,7]. Xu et al. reported a reduced risk of incident type 2 diabetes in patients with OSA treated by CPAP [8]. However, the majority of studies to date were performed in patients with OSA, but not necessarily associated with OHS. Given the high rate of comorbidities in patients with OHS, we perceived a need to evaluate the impact of previous treatment of OSA with a PAP device on the prevalence of comorbidities at the time of diagnosis of OHS.

We hypothesized that previous treatment of OSA with a PAP device before shifting to NIV would decrease cardiometabolic comorbidities at the time of OHS diagnosis. The aim of this study was therefore to evaluate the impact of previous treatment of OSA with a PAP device on the prevalence of comorbidities at the time of OHS diagnosis.

## 2. Patients and Methods

We performed a retrospective, single-center study in a cohort of patients with OHS at the Department of Respiratory Medicine of the University Hospital (CHU) of Martinique from 1 January 2019 to 30 August 2022. The University Hospital of Martinique is the reference center for the island, and the Department of Respiratory Medicine is the only unit specialized in the treatment of respiratory diseases on the island.

Patients with OHS and severe OSA were included in the study. The diagnosis of OHS was based on body mass index (BMI) > 30 kg/m^2^, daytime hypercapnia, and the absence of another cause of hypoventilation. To guarantee this latter criterion, we excluded patients with a smoking history of more than 10 pack-years in women and 15 pack-years in men, with associated obstructive syndrome (FEV1/FVC < 70%) in whom associated COPD could not be ruled out. Severe OSA was defined as a patient with a prior diagnosis of OSA and treated with PAP or a diagnosis of severe OSA (AHI ≥ 30/hour) on polygraphy or polysomnography.

Senior cardiologists assessed the involvement of underlying cardiac disease. Clinical, ECG, echocardiographic and biological abnormalities were used to define congestive heart failure according to recent guidelines [9]. Respiratory diseases were confirmed by a pulmonologist after clinical examination, pulmonary function tests and, if necessary, chest imaging. Acute respiratory failure was defined as the sudden onset of dyspnea requiring attendance at the emergency department, associated with hypoxemia and/or hypercapnia. In patients with OHS, patients had respiratory acidosis.

Diabetic patients were patients with a diagnosis of diabetes with antidiabetic therapy.

We recorded sociodemographic data (age at diagnosis, sex, BMI), medical conditions (diagnostic circumstances, comorbidities at diagnosis, arterial blood gases at diagnosis and spirometry data) for all patients.

The primary outcome was the prevalence of cardiac diseases at the time of OHS diagnosis. Secondary outcomes were diagnostic circumstances (acute respiratory failure or stable state), the prevalence of metabolic diseases (diabetes mellitus) and the prevalence of chronic respiratory diseases (asthma, interstitial lung diseases or other chronic pulmonary diseases) at the time of OHS diagnosis.

The study was performed in accordance with the declaration of Helsinki and received the approval of the Institutional Review Board of the University Hospitals of Martinique (under the number 2022/158). At every follow-up visit, patients received an information letter about the study and had the possibility to explicitly express their opposition to the use of their medical data for routine purposes. In this study, no patient opposed the inclusion of their data in the study.

Descriptive analysis was performed. Numerical variables are described as mean ± standard deviation (SD) and categorical variables as number and percentage. Patients with prior treatment of OSA with a PAP device were compared to those with no history of prior treatment with a PAP device, using the Student *t*-test for continuous variables and the chi-square or Fisher’s exact test for proportions.

Statistical analyses were performed using SAS software release 9.4 (SAS Institute, Inc., Cary, NC, USA). Tests were considered significant for *p*-values less than 0.05.

## 3. Results

We included 97 patients with OHS associated with severe OSA who were followed up in the Respiratory Department between 1 January 2019 and 30 August 2022. There were 44 women (76%) and 23 men (24%). Among them, 54 patients (56%) had previous treatment of OSA with a PAP device before shifting to NIV (PAP group), and 43 (44%) had no previous treatment of OSA with a PAP device before the initiation of NIV (no PAP). The patients’ characteristics and comorbidities are detailed in Table 1.

The prior treatment with a PAP device was associated with a low prevalence of chronic heart failure at the time of OHS diagnosis (relative risk (RR) 0.44, 95% CI 0.23–0.86) and with a diagnosis of OHS against a background of acute respiratory failure (RR 0.60, 95% CI 0.46–0.77).

## 4. Discussion

Previous treatment of OSA with a PAP device before shifting to NIV appears to be associated with a decreased prevalence of heart diseases at the time of OHS diagnosis. Patients with no previous CPAP were more frequently diagnosed with acute respiratory failure. There were more patients with asthma among those who were already on CPAP. Conversely, there was no difference in arterial blood gas and spirometry parameters between those with and those without prior treatment of OSA with a PAP device.

Previous treatment of OSA with a PAP device before shifting to or initiating NIV seemed to reduce the prevalence of heart disease (30% versus 53% (*p* = 0.02)), especially the prevalence of chronic heart failure (18% versus 42% (*p* = 0.01)) and the diagnosis with acute on chronic respiratory failure (56% versus 93% (*p* < 0.0001)) at the time of OHS diagnosis. We did not observe any reduction in hypertension, rhythm disturbances or pulmonary hypertension among those with prior treatment of OSA with a PAP device. We noted above all a lower risk of other heart diseases, notably chronic heart failure, among those with prior treatment of OSA with a PAP device. Many asthma patients had previously been treated with a PAP device at the time of OHS diagnosis, but the difference was not statistically significant. This could be explained by the fact that these patients most often benefit from OSA screening based on respiratory symptoms. In Martinique, OSA is mainly managed by private doctors in community practice, in the absence of a hospital with sufficient capacity. According to the French national statistics institute (INSEE), 53% of the Martinican population was overweight or obese in 2019 [10]. Both OSA and OHS are almost certainly underdiagnosed in Martinique.

Cardiovascular and metabolic comorbidities are a major burden of OHS [11]. Cardiovascular risk in OSA has been widely investigated, and there is an excess risk, reportedly mediated by mechanisms such as neuro-hormonal dysregulation, endothelial dysfunction and inflammation [12]. The main forms of cardiovascular disease observed in patients with OHS include hypertension, heart failure, arrhythmias and coronary artery disease [13]. Previous studies have shown an improvement in hypertension under CPAP, but there have also been reports of improvements in electrophysiological parameters and cardiac biomarkers [6,7,14,15]. In the SAVE study, no reduction in cardiovascular events was found in patients with PAP device treatment compared to those treated with usual care after 3.7 years of follow-up among patients with moderate to severe OSA [16]. The ISAACC study, with 3.35 years of follow-up, and the RICCADSA study, with almost 5 years (57 months) of follow-up, also reached similar conclusions [17,18]. However, PAP therapy does seem to be associated with a reduced risk of heart failure, or an improvement in left ventricular ejection fraction [13]. Most of the prospective studies investigating cardiovascular events were limited to durations of less than 5 years. Although many patients abandon their PAP therapy by 3 years [19], prolonged adherence to PAP therapy could have beneficial effects on the cardiovascular risk profile. Unfortunately, we did not record the duration of prior treatment of OSA with a PAP device before shifting to NIV therapy in our study, but the patients may have had different treatment durations from those reported in prospective studies, which could explain the discrepancies between our findings and previous reports. The difference may also stem from the fact that previous studies were performed in patients with OSA, and not specifically in those with OHS.

We observed no effect of prior treatment of OSA with a PAP device before shifting to NIV on the prevalence of diabetes at the time of diagnosis. In the literature, an increase in the incidence of diabetes has been reported in patients with OSA, but PAP device therapy does not appear to be associated with this risk [4,8], although it has been reported that CPAP improves the HOMA insulin resistance index [20].

The prevalence of asthma in our study population was higher than that in other reports in the literature [21], and the rate was numerically, albeit not statistically significantly, higher among those with prior treatment of OSA with a PAP device. Patients with asthma often have more marked respiratory symptoms and therefore may be more likely to undergo testing for OSA, particularly during contacts with respiratory medicine specialists, and therefore, they may be prescribed PAP device therapy. In our practice, around 24% of the patients with OHS have concurrent asthma. While CPAP improves quality of life and symptoms in asthma patients, its effect on the control and severity of asthma remains debated [22,23].

Our findings support the idea that early treatment with a PAP device in obese patients with severe OSA is associated with a lower prevalence of cardiac diseases at the time of OHS diagnosis.

### Study Strengths and Limitations

Our study is a population-based study with the avoidance of selection bias. This concerns people who are not habitually included in clinical trials on OHS. However, our study has some limitations. Firstly, this was a single-center study, in a homogeneous population. Of note, the population of Martinique is predominantly of Afro-Caribbean descent, an ethnicity not commonly represented in large, multicenter, randomized trials. The results of our study may therefore not be generalizable to populations of other descent. Second, this was a retrospective study, with the potential bias inherent to this type of investigation. Although both groups were comparable in terms of sociodemographic characteristics, the absence of randomization precludes drawing any conclusions of causality and leaves some margin for potential selection bias. Certain data, such as adherence to PAP or the duration of PAP therapy prior to OHS diagnosis, were not recorded but could have provided additional insights. Nevertheless, our results are in line with those of the literature and should prompt us to perform more frequent screening for OSA among obese patients with a view to initiating appropriate therapy if needed.

## 5. Conclusions

Previous treatment of OSA with a PAP device before shifting to or initiating NIV is associated with a reduced prevalence of cardiac diseases at the time of diagnosis of OHS. Early screening and early treatment of severe OSA in obese patients could help to reduce the risk of cardiovascular diseases in this population.

## Figures and Tables

**Table 1:** Patient characteristics at OHS diagnosis.

Characteristics	All	PAP * (*n* = 54)	No PAP (*n* = 43)	*p*-Value
Mean age at OHS diagnosis (years)	97	67 ± 16	66 ± 15	0.7
Charlson comorbidity index	77	5 ± 2	5 ± 2	0.6
Number of comorbidities		4 ± 1	4 ± 2	0.4
Female sex	74	40 (74)	34 (79)	0.6
Age ≥ 70 years (%)	45	24 (44)	21 (49)	0.6
Body mass index (BMI) ≥ 40 (kg/m^2^) (%)	50	27 (50)	23 (53)	0.9
**Comorbidities**				
Heart diseases (%)	39	16 (30)	23 (53)	**0.02**
Cardiac arrhythmia (%)	20	9 (17)	11 (26)	0.3
Pulmonary hypertension (%)	11	5 (9)	6 (14)	0.4
Chronic heart failure (%)	28	10 (19)	18 (42)	**0.01**
Arterial hypertension (%)	82	46 (85)	36 (84)	0.8
Peripheral arterial disease (%)	08	4 (7)	4 (9)	0.7
Diabetes mellitus (%)	60	31 (57)	29 (67)	0.3
Asthma (%)	28	20 (37)	8 (19)	0.07
**Circumstances of diagnosis of OHS**				
Acute respiratory failure (%)	70	30 (56)	40 (93)	**<0.0001**
Follow-up of sleep apnea (%)	19	19 (35)	0 (0)	
Unknown (%)	07	5 (9)	2 (5)	
**Arterial blood gas**				
pH		7.35 ± 0.05	7.34 ± 0.06	0.3
pCO2 ^α^ (mmHg)		58 ± 12	58 ± 11	0.9
pO2 ^β^ (mmHg)		65 ± 13	64 ± 14	0.7
Bicarbonates (mmol/L)		31 ± 6	30 ± 6	0.4
**Pulmonary function tests**				
FEV1 ^γ^ (% of theoretic value)		59 ± 20	54 ± 16	0.2
FEV1/FVC		80 ± 16	81 ± 11	1
FVC ^δ^ (% of theoretic value)		62 ± 19	55 ± 17	0.1
TLC ^ε^ (% of theoretic value)		72 ± 16	68 ± 14	0.3
RV ^µ^ (% of theoretic value)		96 ± 33	94 ± 26	0.9
ERV ^§^ (% of theoretic value)		68 ± 52	60 ± 39	0.4

* Continuous positive airway pressure, ^α^ PaCO2: partial pressure of carbon dioxide, ^β^ PaO2: partial pressure of oxygen, ^γ^ forced expiratory volume in 1 s, ^δ^ forced vital capacity, ^ε^ total lung capacity, ^µ^ residual volume, ^§^ expiratory reserve volume.

## Data Availability

The datasets used and/or analyzed during the current study are available from the corresponding author on reasonable request.

## Abbreviations

BMI	body mass index
CHU	university hospital center
CPAP	continuous positive airway pressure
COPD	chronic obstructive pulmonary disease
ERV	expiratory reserve volume
FEV1	forced expiratory volume in 1 s
FVC	forced vital capacity
mmHg	millimeter of mercury
NIV	non-invasive ventilation
PaCO2	partial pressure of carbon dioxide
PAP	positive airway pressure
PaO2	partial pressure of oxygen
OHS	obesity-hypoventilation syndrome
OSA	obstructive sleep apnea
RV	residual volume
TLC	total lung capacity
